# An Unusual Representation of an Odontogenic Cyst Engulfing the Inferior Alveolar Nerve

**DOI:** 10.7759/cureus.31563

**Published:** 2022-11-16

**Authors:** Raid Khayat, Maisa O Al-Sebaei

**Affiliations:** 1 Oral and Maxillofacial Surgery, King Abdulaziz University, Jeddah, SAU

**Keywords:** enucleation, jaw lesions, mandibular canal, inferior alveolar nerve, odontogenic cyst

## Abstract

This report investigates the growth of an odontogenic cyst around the inferior alveolar nerve (IAN), a crucial vital structure to be approached during oral and maxillofacial surgical procedures. A unique aspect of this case was that the cyst completely engulfed the IAN rather than displacing it and left the patient asymptomatic. Under general anesthesia, complete enucleation of the cyst was performed while the IAN was dissected away. The patient had a temporary neurosensory dysfunction, which they fully recovered from. It is extremely rare for pathological entities to engulf the mandibular canal (MC); however, if the lesion is noninvasive, an excellent prognosis is likely to be expected.

## Introduction

The intervention of oral pathologic lesions can range from minor to major according to the diagnosis. The management of intraosseous lesions is particularly complicated because of their limited accessibility. Moreover, the presence of adjacent vital structures requires decisions that may be challenging to the clinician as well as the patient. With this in mind, impacted lower third molars are associated with a higher risk of developing several types of pathological conditions.

These include lesions that are present as unicystic, well-defined radiolucencies in the posterior mandible, such as dentigerous cysts, unicystic ameloblastomas, and odontogenic keratocysts. Their association with an impacted tooth is reported to be 65%, 50%-80%, and 27%, respectively, predominantly with a third molar [1]. The prevalence of radicular cysts associated with impacted third molars is estimated to be 4.7% [2]. Other, less prevalent lesions, such as calcifying cystic odontogenic tumors, may also develop together with impacted lower third molars [3]; however, radiographically, they appear as a mixed radiolucent-radiopaque lesion [1].

Furthermore, mandibular intraosseous pathology that is associated with the inferior alveolar nerve (IAN) is infrequently seen; nevertheless, some cases have been reported. These may include developmental cysts [4], benign osteogenic tumors [5], benign neural-derived tumors such as schwannomas [6,7], and perineuriomas [8,9].

## Case presentation

**Figure 1:**
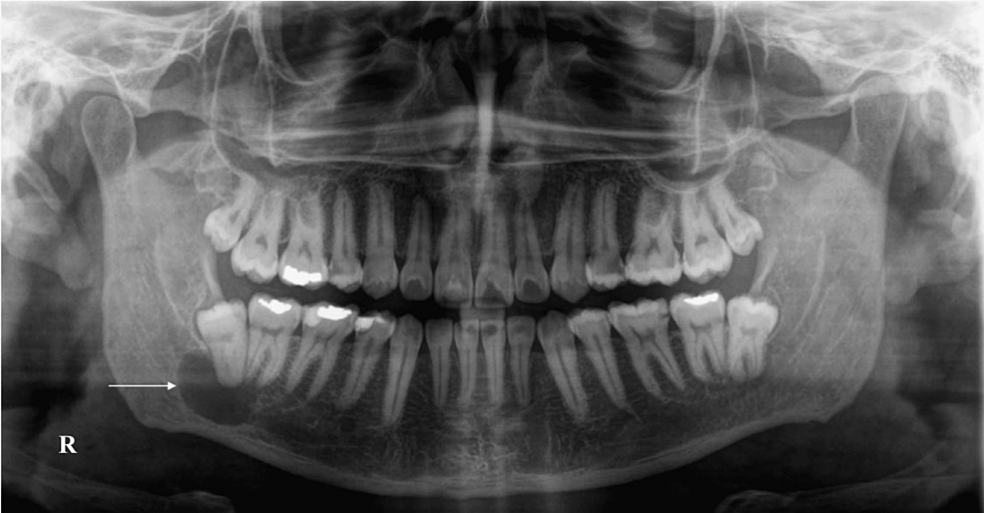
Preoperative orthopantomogram. The radiograph shows a unilocular radiolucent lesion that is related to the lower right molar area and is superimposed on the mandibular canal (white arrow; R, right side).

A 30-year-old Middle Eastern man was referred to the oral and maxillofacial surgery department at King Abdulaziz University after a radiolucent lesion was identified by the dentist on the orthopantomogram (OPG) radiograph during a routine examination (Figure 1). The patient is healthy and does not have any allergies or significant medical history. As for his oral condition, the patient reported no symptoms. The extraoral examination indicated no evidence of lymphadenopathy, swelling, or asymmetry. Intraoral examination revealed mild lingual alveolar bone expansion with focal areas of palpable cortical resorption. The lower right first and second molars were vital and responded normally to cold testing, but the lower right third molar was covered by soft tissue and inaccessible for the same examination. Also, there was no altered sensation of the lower lip and chin on the ipsilateral side.

Upon OPG interpretation, a well-defined, noncorticated radiolucency was seen in the right posterior mandible and inferior to the molars. The lesion extends from the mesial root apex of the second molar to the distal area of the third molar. It also communicates with the follicular space of the third molar, whereas there is no radiographic effect on the first molar. The mandibular canal (MC) begins to expand as it enters the lesion, and then its cortical plates cannot be seen, suggesting either superimposition or engulfment.

**Figure 2:**
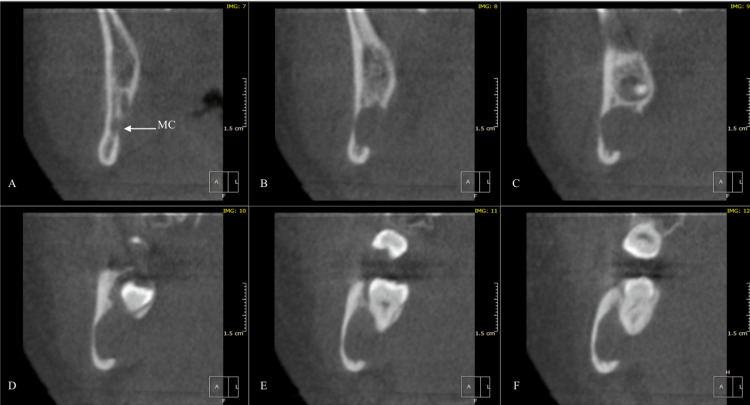
Preoperative cone-beam computed tomography (coronal view). (A) MC. (B-F) Consecutive cuts show the expansion of the radiolucent lesion, with the MC disappearing inside it. Also, there is focal resorption of the corresponding lingual cortical bone and thinning of the buccal cortex. MC, mandibular canal

**Figure 3:**
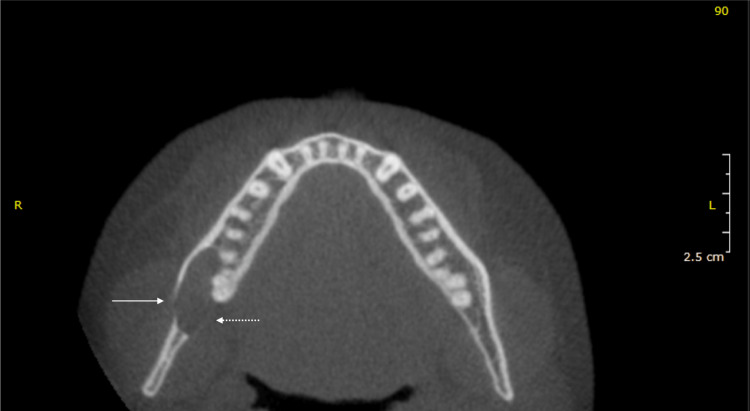
Preoperative cone-beam computed tomography of the mandible (axial view). The axial cut is at the level of the third and second molar-root apexes. The white arrow points to the right mandibular lesion. The dotted arrow points to the resorbed cortical bone on the lingual side.

Cone-beam computed tomography has confirmed the presence of the IAN within the lesion (Figure 2). Also, areas of perforations in the mandibular lingual cortical plate and thinning of the buccal cortical plate were clear on the images (Figure 3). Taking into account these findings, there is a possibility of the existence of a benign tumor of approximately 21 mm × 14 mm × 9 mm in size. The consulting radiologist suggested a neural-derived tumor as the top differential diagnosis, followed by a unicystic ameloblastoma.

Extraction of the third molar with an incisional biopsy was performed through a buccal window as part of the surgical plan. In the microscopic examination, a cystic process was lined by a thin, stratified squamous epithelium with a focal area of thickening. There was a patchy chronic inflammatory infiltrate and cholesterol clefts in the cystic fibrous connective tissue wall, and the diagnosis was an odontogenic cyst. An examination of the whole specimen following surgical removal was required as there were no characteristics of any particular odontogenic cyst found on the biopsy. Based on that, the surgical plan was to proceed with enucleation under general anesthesia.

The risk of IAN injury and its consequences were discussed thoroughly with the patient. A full mucoperiosteal flap was raised, and a large buccal bony window was opened against the lesion’s site. The cyst was dissected gently from the nerve in multiple fragments until the entire area looked clear of any detectable remnants. There was no severance or direct injury to the IAN. Using a rotary instrument, peripheral ostectomy was done to the surrounding internal surface of the mandible, and the flap was sutured in primary closure. In addition to standard postoperative instructions, a soft diet was advised to avoid an unlikely but potential mandibular fracture.

**Figure 4:**
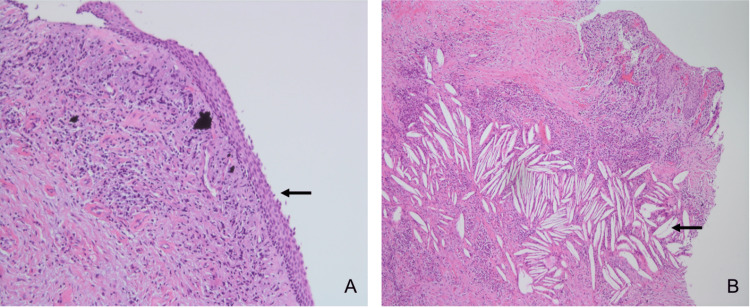
Histologic sections of the excisional biopsy. (A) The histologic sections revealed nonkeratinized stratified squamous cystic epithelial lining (black arrow). (B) The cystic fibrous connective tissue stroma is infiltrated by mixed inflammatory cells and abundant cholesterol clefts (black arrow; hematoxylin and eosin stain, 4×).

Histologically, the excisional biopsy did not exhibit any distinctive characteristics for a specific odontogenic cyst. The cystic fibrous connective tissue wall showed patchy inflammatory infiltrate, cholesterol cleft, and giant cell reaction (Figure 4). The final histopathologic diagnosis was an *odontogenic cyst with a giant cell reaction*.

Follow-up

**Figure 5:**
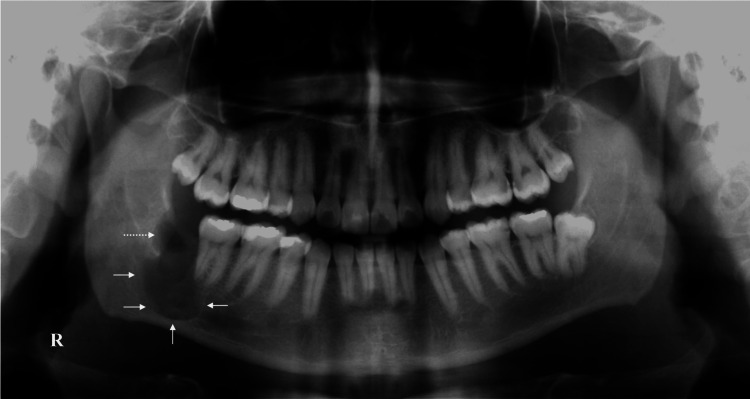
A panoramic view at the one-week post-excisional biopsy and peripheral ostectomy (white arrows). The dotted arrow points to the socket of the previously extracted lower right third molar (R, right side).

Clinical examination of the surgical site one week after surgery found no erythema or signs of infection. A radiographic examination showed that the defect was large and the lower border of the mandible was intact (Figure 5). The only complaint the patient had was lower lip numbness on the right side, which was predicted ahead of the surgical intervention. At the two-week follow-up, the wound healed, the postoperative pain subsided, extraoral edema significantly decreased, and the maximal incisal opening was normal. During the first, second, and sixth weeks after surgery, the IAN injury was evaluated through nerve mapping and testing pain, touch, and two-point discrimination to evaluate its improvement, which showed continued but slow progress.

It was observed that as time goes by, it can be difficult to assess the symptoms of the nerve injury as the patient may have adapted to the sensation and may have trouble describing it accurately. After six months, the IAN function was fully restored; the numbness subsided spontaneously without any pharmaceutical intervention.

**Figure 6:**
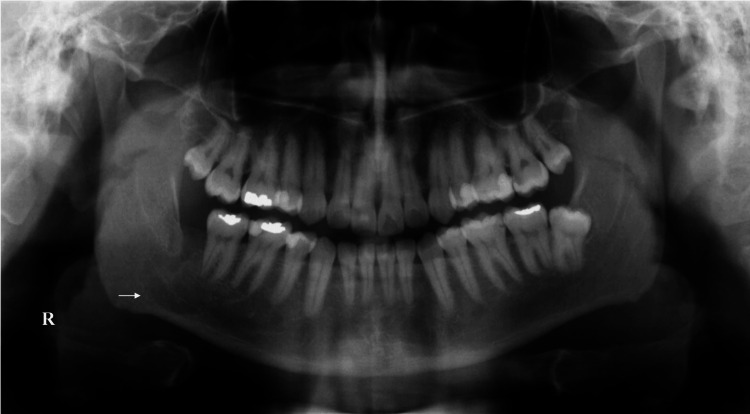
A panoramic view at the one-year follow-up. The X-ray shows a significant bone refill (white arrow; R, right side).

At the one-year follow-up, OPG shows significant yet unfinished bone deposition, which is indicative of the size of the defect; also the MC can be traced within the enucleated lesion’s region (Figure 6). Furthermore, there has been no discernible recurrence of the lesion, and the patient will be monitored for another year.

## Discussion

The atypical growth behavior of the lesion around the IAN radiographically and the masking effect of the inflammatory infiltrates on the histopathological sections have caused a diagnostic dilemma. However, the incisional biopsy ruled out the differential diagnosis of tumors and identified an odontogenic cyst, so the surgical intervention of choice was enucleation and peripheral ostectomy.

Some of the histopathology features of the excisional biopsy are consistent with those of a periapical cyst or an inflamed dentigerous cyst, both of which account for the majority of jaw lesions [10]. Routinely, a correlation of the lesion with its radiographic features is recommended to aid in the diagnosis. However, the behavior on the radiograph is uncharacteristic. Aside from its resorbing effects on the buccal and lingual cortices, the association with the IAN is atypical of the presentation of odontogenic cysts.

Literature has revealed frequent types of intraosseous lesions that are involved with the IAN. There has been a correlation between the direction of the MC displacement and various types of pathological entities. Odontogenic tumors such as ameloblastomas [9,10] and myxomas can displace the MC inferiorly and buccally, whereas vascular lesions have been found to push the MC lingually [11]. The MC was also found to be displaced inferiorly by odontogenic cysts as well, such as radicular cysts, odontogenic keratocysts, and dentigerous cysts [12].

Regarding preoperative sensory dysfunction of the IAN, a dentigerous cyst was reported to displace the MC inferiorly and cause paresthesia [13]. In a similar behavior, a radicular cyst has displaced the MC inferiorly, causing a narrowing of the canal, which resulted in the loss of touch and sensation [14]. In contrast, a rare case of a unilateral enlargement of almost the entire length of the MC has been attributed to a noninvasive radicular cyst [15]. A more aggressivepattern of sensory dysfunction is where an odontogenic keratocyst displaces the MC inferiorly and causes perforation of the upper cortex of the canal, triggering neuropathic pain that is diagnosed as trigeminal neuralgia [16].

However, a rare case of a large dentigerous cyst associated with an impacted lower third molar was reported to surround the IAN based on 3D radiographic images [4]. It was found that the characteristics of this dentigerous cyst closely resembled our reported case in terms of buccolingual expansion, cortical thinning, and engulfment of the IAN without invasion. The association with the lower impacted third molar was also mutual but not with the crown of the tooth as a characteristic pattern of dentigerous cysts. To our knowledge, this presentation of an odontogenic cyst engulfing the IAN and confirmed by cone-beam computed tomography is the second reported case in the literature.

The sensory function of the IAN and the morphology of the MC have been reported to be affected by neural origin lesions such as intraosseous neurofibroma [17] and intraosseous intraneural perineurioma [8]. Conversely, IAN-derived schwannomas were reported to be asymptomatic; however, the borders of the MC were not detectable within the region of the lesion on the OPG [18,19], which was also mutual with our case.

In this report, the neurosensory disturbance developed postoperatively was caused by the dissection of the cyst from the IAN. Although the nerve as a whole was intact, a potential minor injury could have occurred. Approximately six months after the surgical intervention, the patient regained normal sensation and, therefore, could be categorized as having axonotmesis based on the Seddon classification [20].

## Conclusions

Preoperative radiographic differential diagnosis of lesions involved with the IAN is quite challenging. The unique presentation of an odontogenic cyst engulfing the IAN seems to have a conservative effect that does not produce preoperative neurosensory dysfunction compared to nerve displacement or compression. Its intraosseous position and challenging accessibility do not preclude a positive prognosis. The IAN was not damaged and gained full recovery primarily because of the noninvasive nature of the lesion and the cautious operative technique.
